# Implementation strategies for the patient safety reporting system using Consolidated Framework for Implementation Research: a retrospective mixed-method analysis

**DOI:** 10.1186/s12913-022-07822-9

**Published:** 2022-03-28

**Authors:** Daisuke Koike, Masahiro Ito, Akihiko Horiguchi, Hiroshi Yatsuya, Atsuhiko Ota

**Affiliations:** 1 Department of Public Health, Fujita Health University School of Medicine, Toyoake, Aichi Japan; 2 Department of Gastroenterological Surgery, Fujita Health University School of Medicine Bantane Hospital, Nagoya, Aichi Japan; 3 Department of Public Health and Health Systems, Graduate School of Medicine, Nagoya University, Nagoya, Japan

**Keywords:** Implementation science, Patient safety, Safety management, Risk management, Japan

## Abstract

**Background:**

Healthcare-related adverse events occur because of complex healthcare systems. The patient safety reporting system is a core component of patient safety initiatives in hospitals. However, hospital management often encounters a cultural barrier with its implementation and struggles to overcome the same. Implementation science would be useful for analysing implementation strategies. This study determines the effects of the implemented strategy on an increase in the number of patient safety reports and the determinants of successful implementation, using the implementation framework.

**Methods:**

Mixed method analysis was performed in Fujita Health University Hospital (FHUH), a large volume hospital in Japan. We identified strategies to implement the patient safety reporting system by scrutinising internal documents using the Consolidated Framework for Implementation Research (CFIR). The electronic reporting systems developed in 2004 in the FHUH and the number of reports were analysed using the staff data and hospital volumes.

**Results:**

Reports (*n* = 110,058) issued between April 2004 and March 2020 were analysed. The number of reports increased from 2004 to 2008 and from 2013 to 2019, reaching 14,037 reports per year. Between 2009 and 2012, the FHUH experienced a stagnation period where the number of reports were not increasing. From the qualitative materials, we identified 74 strategies which contributed to the implementation of the patient safety reporting system. Among these, the domain of ‘intervention characteristics’ in the CFIR contained 12 strategies, ‘outer settings’ contained 20, ‘inner settings’ contained 21, ‘characteristics of individuals’ contained 8, and ‘process’ contained 13. There were two concentrated periods of the implemented strategies, the number was 17 in 2007 and 10 in 2016. These concentrated periods preceded a remarkable increase in the number of patient safety reports.

**Conclusions:**

A safety culture had been fostered in FHUH in the study period. A relationship between number of strategies and development of a reporting culture was observed. The intensity of adequate strategies was needed for implementation of patient safety reporting system. Therefore, the implementation framework is useful for analysing patient safety initiatives for safety culture.

**Supplementary Information:**

The online version contains supplementary material available at 10.1186/s12913-022-07822-9.

## Background

Patient safety is now a significant aspect of healthcare quality in many countries. Healthcare-related patient harm can occur in any healthcare setting. It occurs because of an incomplete healthcare system, and not due to poor performance [1]. Healthcare systems are regarded as complex systems where healthcare workers are often unable to predict how the systems work based on their knowledge of each component, because of too many interactions [2]. The healthcare system is composed of many stakeholders who have certain responsibilities for patient safety. With the involvement of various stakeholders, healthcare systems develop into complicated systems to provide high-quality medical services. However, a complicated system could have an opposite effect, causing patient harm. Unfortunately, many painful and harmful, but preventable accidents have occurred in the healthcare system due to its incomplete and complicated nature. For example, wrong patient surgery, wrong-side surgery, an adverse effect caused by inappropriate use of a drug, or severe allergy caused by a known allergic substance [3, 4]. Previous studies from Japan and the U.S. [1, 5] indicate significantly high rates of mortality caused by healthcare-related adverse events.

To achieve patient safety in such a complex system, we must learn from our experience with wrong events [6]. The patient safety reporting system is a core component of patient safety initiatives. It is a learning system for healthcare institutions to improve healthcare systems and enhance patient safety. An incident reporting system was first developed in other industries, such as aviation, and was later introduced into healthcare. In 1999, the Institute of Medicine reported that error and incident reporting systems are key strategies to learn from incidents and prevent their recurrence [1]. Institutes and organisations can identify problems occurring in clinical settings through the reporting system and provide feedback with some countermeasures.

A patient safety reporting system is implemented in most institutions in both developing and developed countries. Although it seems easy for institutional executives to superficially implement a reporting system in their institutions, its complete implementation and establishment as a learning system is still a significant challenge [7]. Institutional executives and care providers face several barriers when employing a reporting system. For example, fear of blame, the dysfunctional system of reporting, insufficient knowledge or skills to report, report-hesitant work environment, lack of feedback and communication in response to reporting, and the absence of positive reporting culture [8, 9]. Hence, systematic approaches are necessary to overcome these barriers and implement the reporting system thoroughly in the institution.

To overcome the discrepancy between policy and practice, and to promote the systematic uptake of the policy into routine practice, implementation science and framework concept was developed in healthcare recently [10–12]. Integration of strategies to improve reporting in an existing system was part of implementation of patient safety. The Consolidated Framework for Implementation Research (CFIR) was developed in implementation science to analyse the implementation strategies [11]. The theoretical implementation framework enables us to analyse, interpret, and understand why innovation is successfully implemented [11–13]. CFIR is used through pre-, during, and post-implementation phases. In the pre-implementation phase, the CFIR provides a list of well-defined constructs. This allows a leader of initiatives or a researcher to assess potential weaknesses and refine the implementation strategy for a full-scale implementation. In the post-implementation phase, the CFIR can be used to explore the relationship between strategies and outcomes of implementation. The researcher can identify factors that influence the outcomes, using CFIR. It has already been applied in clinical medicine and public health [14, 15]. However, few studies have applied implementation science to the analyses of patient safety initiatives.

In Fujita Health University Hospital (FHUH), a verifiable patient safety reporting system was implemented in 2004, and the number of reports have been increasing with many implementation strategies being introduced by the safety department. The annual number of safety reports reached around 14,000 in 2019, which appears an indication of established safety culture. This study aims to investigate the relationship between the implemented strategies and the increase in patient safety reports. Moreover, this study aims to reveal the determinants of successful implementation of the patient safety reporting system.

## Method

### Study setting

This study was conducted in FHUH, a tertiary academic hospital in Japan, from April 2004 to March 2020. The hospital serves all types of acute and emergency care with several specialised intensive care units, such as coronary, stroke, and trauma. The total number of beds in the hospital is approximately 1400. Electronical reporting systems at this institution were developed in 2004.

### Study design

This study utilised a mixed-methods approach with quantitative and qualitative data. We analysed the hospital’s management system for patient safety by focusing on the reporting system and safety department. Additionally, internal documents collected from the hospital’s database were scrutinised.

### Patient safety reporting system

The number of patient safety reports was collected from the hospital’s electronical reporting system from April 2004 to March 2020. Various hospital professionals had an account of the reporting system: physicians, nurses, pharmacists, laboratory technicians, radiological technicians, medical engineers, therapists of rehabilitation, administrators, and others. The reporting system was voluntary from the beginning till the end of the study period and secured the confidentiality of reporters.

### Quantitative data source of the hospital volume

The numbers of staff in each profession and patient admissions were collected from the hospital annual reports issued every six months during the study period. 

### Qualitative data source of safety management

We reviewed 631 internal document records issued during the study period. Documents and materials of the hospital safety board (*n* = 105), with top management and safety committee meetings (*n* = 82), were counted. The discussion record of the board (*n* = 156) and committee (*n* = 164) were also analysed. We also collected 124 in-hospital newspaper, published monthly by the safety department to all clinical staff as ‘safety news’. We referred the outer materials such as some ordinances and press reports that were mentioned in the internal document source to complement the qualitative data.

### Implementation framework

We used the CFIR to analyse our implementation strategies, which provides a menu of constructs associated with effective implementation [11]. The CFIR has five domains containing a wide range of implementation strategies: intervention characteristics, outer setting, inner setting, characteristics of individuals, and process. These domains contain eight, four, fourteen, five, and eight constructs, respectively. A detailed description of each construct is available on the CFIR webpage [16]. DK reviewed all study sources and identified the strategies. These strategies were classified into constructs according to their definitions. AO verified and confirmed the classification of strategies. Disagreements were resolved by consensus between DK and AO.

## Results

### Annual data of the hospital and patient safety reports

**Fig. 1:**
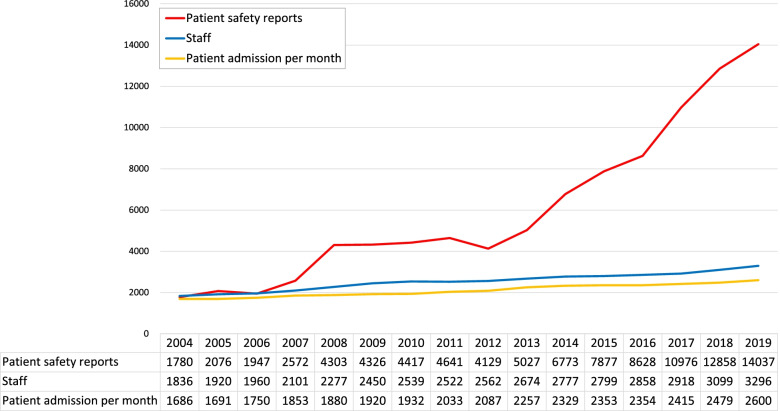
Number of patient safety reports, staff, and patient admission in Fujita Health University Hospital

The number of staff were 1836 in 2004 and continued to increase during the study period. It reached 3296 in 2019, about 1.8 times of 2004 (Fig. 1). The number of patient admissions also increased from 1700.7 patients per month to 2615.0 in fiscal 2019. From April 2004 to March 2020, a total of 110,058 reports were collected from the electronic reporting system. Duplications were excluded and 96,512 reports were analysed in this study. The number of reports gradually increased from the first implementation, in 2004, to 2008 (Fig. 1) and stagnated from 2008 to 2012. An increment was observed again after 2013, and the number of reports reached approximately 14,000 in 2019.

### Implementation strategies

**Fig. 2:**
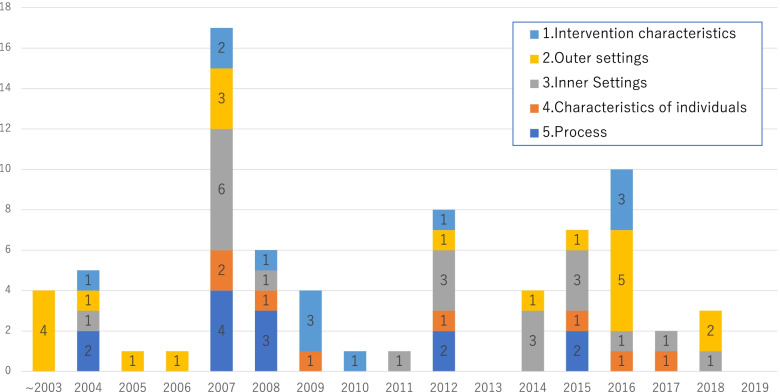
The number of strategies identified using the consolidated framework for implementation research (CFIR)

We identified 74 strategies from the qualitative materials and classified them using the CFIR (Table 1). There were four ‘outer settings’ strategies issued before the study period and were identified from internal documents. We included them since they had a strong and continuous effect on the implementation of the reporting system during the study period. Of the 74 strategies, 12 corresponded to ‘intervention characteristics,’ 20 to ‘outer settings,’ 21 to ‘inner settings,’ 8 to ‘characteristics of individuals,’ and 13 to ‘process.’ Some of these strategies were important in developing the safety culture, as described in detail below. Figure 2 shows the annual number of implementation strategies. There were two concentrated periods in the number of implemented strategies during the study period: 2007 and 2016.

### Intervention characteristics

Among the 12 strategies classified as ‘intervention characteristics,’ ‘staffing the deputy hospital director as the director of the safety department’ was classified as an intervention source, as they are an important resource of the hospital executive management system. This reflects the priority of the safety department given by hospital management. Several revisions of the patient safety report forms were made so that the frontline staff could report easily and overcome the barrier of complexity. Thus, these revisions were classified as a complexity. Five strategies were identified as relative advantages. Recommendations to report were repeatedly announced by the safety department to frontline staff without a blame policy. The safety department explicitly showed safety policy not to think that reporting was a disadvantage for frontline staff.

### Outer settings

‘Outer setting’ strategies were mainly provided by the government and external authentication. FHUH is one of 87 special functioning hospitals designated by the Ministry of Health, Labour and Welfare, Japan (MHLW). This directly affected our safety management policy. FHUH obtained two external authentications during the study period: one was a certification from the Japan Council for Quality Health Care (JCQHC), and the other was a certification from Joint Commission International (JCI). The establishment of a safety management system was mandatory in the requirements of the JCQHC, and the quality management system was included in the JCI. In Japan, there were two severe medical incidents that the media largely reported on. First, misidentification of patients and wrong injection of disinfectors in large-scale hospitals in 1999 [17]. Second, the high mortality rate of a specific surgery that occurred in two large-sized hospitals in 2014, similar to the Bristol heart scandal in England [18–20]. These evoked the public movement for patient safety and the government’s requirement for special functioning hospitals in Japan.

### Inner settings

The ‘inner setting’ strategies reflected the policy of the top management and safety departments of the institution. The hospital management prioritised staff in the safety department. Top management resources priority and staff in the safety department. Nurses, pharmacists, physical therapists, and physicians have been full-time staff in the department, antecedent of government requirements. The safety department implemented various implementation strategies. Training regarding patient safety was the input for all clinical staff. Moreover, labour costs of reporting decreased with the revision of the report system. Recommendation to report, the annual award of the report, and the Good Job report in the reporting system encouraged frontline staff to report. Most strategies in the ‘inner settings’ were classified into an ‘implementation climate’.

### Characteristics of individuals

Eight strategies were classified as ‘characteristics of individuals.’ The safety department implemented such strategies as the annual award of reporting, safety newspaper, and incident investigation to enhance the staff’s perception of receiving feedbacks from the safety department or hospital management. The incident investigation system was performed not only by the safety department but also by other stakeholders, including frontline staff. It was classified into this construct because the involved staff experienced self-efficacy regarding patient safety initiatives.

### Process

In the ‘process’ construct, the ‘availability of the reporting system’ was identified as a strategy. All frontline staff members could easily access the reporting system as the account to login to the reporting system was common with that of the electronic medical record system. Several revisions of the reporting system helped the staff to report incidents. The annual reporting award was also identified as the ‘champion’ in the ‘process’ construct because it could draw the attention of the large-sized hospital in this study. ‘Staffing deputy hospital director as the director of the safety department’ was considered as ‘formally appointed internal implementation leaders’ in this construct and was also classified as ‘intervention characteristics’ as described above.

## Discussion

This study demonstrated that 74 strategies were implemented, aiming at and resulting in a remarkable increase in the number of patient safety reports. The significant increase indicates that a safety culture had been fostered in the hospital. The relationship between the number of strategies and the development of a reporting culture was observed. The increase in reports was much larger than the changes in the number of staff and patients. The safety culture established in the hospital in this study was considered to be adequate and sustainable. A significant increase in the number of reports during a long-term period, was observed in this study. Although such findings have been rarely reported before, a similar experience of fostering a safety culture has been reported in 2020 from Japan [21].

Two periods were characterised with high implementation intensity of the multiple strategies that preceded the subsequent remarkable increase in the number of reports. The first concentrated period came in 2007 and the second did in 2016. Previous studies from Western countries and the U.S. have indicated an increase in the number of reports using their initiatives [22–24]. However, their initiatives were time consuming (months or years) from interventions to achieve better outcomes. These initiatives showed that well-constructed strategies would mature the safety culture within a few years and lead to an increasing number of patient safety reports. Therefore, identified strategies preceding the remarkable increase in reports, resulting in a cultural change in the institution, were considered to have fostered a safety culture in the institute. The CFIR can be analysed or used to assist our evaluation of a series of strategies implemented in the patient safety learning system.

The momentum of change in the organisation is essential to foster the safety culture and is defined as a ‘tension for change’ in the ‘inner setting’ of the CFIR [11]. Although it is difficult to specify strategies that were independently effective for the improvement in our study, previous studies have revealed that multiple intervention strategies were effective in the implementation of the patient safety reporting system [22–26]. In our study, synchronised strategies were found in both the ‘inner-’ and ‘outer settings’ domains in 2007 and 2015–2016. This suggests that hospital management handled the outer policy as a chance to enhance the structure of the institution. They effectively resulted in the implementation of patient safety reports. The timely and concentrated implementation of strategies was effective in creating tension for change and could break the barriers and result in the establishment of the culture.

In our experience, there was a stagnation period, between 2009 and 2012, where the number of patient safety reports were not increasing. A few strategies were identified during this period, and safety management appeared to be on the plateau of implementation. However, there is another perspective on stagnation. Since some strategies were expected to exert not only a short-term effect (lasting around a single year, for example) but also a long-term effect to enhance reporting attitude, the observed stagnation could be considered as a period of cultural maturity. For example, training frontline staff about safety culture has an immediate and direct effect on reporting attitude, and policy change or revision of the report format may require a certain period to achieve an obvious effect. It was difficult to determine whether the period observed in this study was essential to foster the safety culture of the institution. Therefore, further study is necessary to confirm this idea, which would be useful to make the healthy reporting culture and foster the safety culture in healthcare institutions.

According to the CFIR, in our study, the largest domain was ‘inner settings’, with 21 strategies. These were classified into ‘culture,’ ‘implementation climate,’ and ‘relative advantage’ constructs of the CFIR. The hospital management and safety department implemented many strategies to mitigate blame culture and foster the reporting culture. The hospital faced many barriers when developing a reporting attitude and a safety culture, with the strongest and persisting barrier being ‘fear of blame’ [8, 23]. The Good Job report and annual award of reporting also encouraged frontline staff to report, since they reflected positive aspects of patient safety reporting [23]. The results of the framework analysis suggested that our implementation strategies were adequate compared with those of previous studies.

In this study, CFIR enabled effective detection of implementation strategies. Many healthcare institutes now implement reporting systems. However, a standard method for assessing the implementation strategy has not been established. The focus of patient safety and quality improvement initiatives is not whether one strategy is correct, but which strategy should be implemented in the current situation [27]. A well-defined framework such as the CFIR would help the patient safety initiative leader to analyse strategies and develop the next implementation strategies. The present study is the first to analyse strategies of patient safety learning systems using the CFIR and reveal the usefulness of the framework in analysing patient safety initiatives.

### Strength and limitations

A strength of the present study is that we well analysed the implementation of patient safety reporting system in FHUH by using the CFIR framework. At the same time, this study had some limitations. First, fewer strategies were identified as ‘characteristics of individuals’ than other domains. We faced a difficulty in identifying the individual-related construct because the study sources were documented materials and were reviewed retrospectively. There were very few documentations referring to the characteristics of individuals, i.e., hospital staff. Second, only recorded documents were collected. There must be many off-the-record practices for patient safety initiatives in clinical situations. However, these unrecorded or unpublished qualitative data were rarely identified in this study. Different approaches from frameworking, such as text mining, may make it possible to further examine the quality and types of patient safety reports. Prospective analysis with patient safety initiatives focusing on implementation science could reveal more clinical strategies.

## Conclusion

This study demonstrated the successful implementation of the patient safety reporting system. The number of reports dramatically increased in this longitudinal study. The intensity of multiple strategies corresponding to implementation science was needed to overcome the fear of blame and foster a safe culture. CFIR was useful for analysing the patient safety initiatives of the reporting system.

## Supplementary Information

**Additional file 1.** Strategies identified for patient safety reporting system using the consolidated framework for implementation research

## Data Availability

All data generated or analysed during this study are included in this published article.

## Abbreviations

U.S: United States
CFIR: Consolidated framework for implementation research
FHUH: Fujita Health University Hospital
MHLW: Ministry of Health, Labour and Welfare
JCQHC: Japan Council for Quality Health Care
JCI: Joint Commission International
